# Estimating cardiovascular risk in patients with type 2 diabetes: a national multicenter study in Brazil

**DOI:** 10.1186/1758-5996-1-22

**Published:** 2009-10-27

**Authors:** Marilia B Gomes, Daniel Giannella-Neto, Manuel Faria, Marcos Tambascia, Reine M Fonseca, Rosangela Rea, Geisa Macedo, João Modesto-Filho, Helena Schmid, Alcina V Bittencourt, Saulo Cavalcanti, Nelson Rassi, Hermelinda Pedrosa, Sergio A Dib

**Affiliations:** 1 Diabetes Unit. State University of Rio de Janeiro, Rio de Janeiro, Brazil; 2 Laboratory for Clinical and Experimental Gatroenterology - LIM07. Hospital das Clínicas. University of São Paulo Scholl of Medicine, São Paulo, Brazil; 3 Division of Endocrinology, Federal University of Maranhão, São Luiz, Brazil; 4 Division of Endocrinology, State University of Campinas, Campinas, Brazil; 5 Centro de Estudos de Diabetes e Endocrinologia do Estado da Bahia (CEDEBA), Salvador, Brazil; 6 Division of Endocrinology, Federal University of Parana, Curitiba, Brazil; 7 Division of Endocrinology, Hospital Agamenon Magalhães, Recife, Brazil; 8 Posto de Assistência Médica de Jaguribe, João Pessoa, Brazil; 9 Division of Endocrinology, Santa Casa de Porto Alegre, Porto Alegre, Brazil; 10 Division of Endocrinology, Instituto de Assistência e Previdência do Servidor do Estado da Bahia, Salvador, Brazil; 11 Diabetes Unit, Santa Casa de Belo Horizonte, Belo Horizonte, Brazil; 12 Division of Endocrinology, Hospital Geral de Goiânia, Goiás, Brazil; 13 Secretaria Municipal de Saúde de Brasília, Brasília, Brazil; 14 Division of Endocrinology, Federal University of São Paulo, São Paulo, Brazil

## Abstract

**Abstract:**

According to Brazilian National Data Survey diabetes is the fifth cause for hospitalization and is one of the ten major causes of mortality in this country.

**Aims:**

to stratify the estimated cardiovascular risk (eCVR) in a population of type 2 diabetics (T2DM) according to the Framingham prediction equations as well as to determine the association between eCVR with metabolic and clinical control of the disease.

**Methods:**

From 2000 to 2001 a cross-sectional multicenter study was conducted in 13 public out-patients diabetes/endocrinology clinics from 8 Brazilian cities. The 10-year risk of developing coronary heart disease (CHD) was estimated by the prediction equations described by Wilson et al (Circulation 1998). LDL equations were preferably used; when patients missed LDL data we used total cholesterol equations instead.

**Results:**

Data from 1382 patients (59.0% female) were analyzed. Median and inter-quartile range (IQ) of age and duration of diabetes were 57.4 (51-65) and 8.8 (3-13) years, respectively without differences according to the gender. Forty-two percent of these patients were overweight and 35.4% were obese (the prevalence of higher BMI and obesity in this T2DM group was significantly higher in women than in men; p < 0.001). The overall estimated eCVR in T2DM patients was 21.4 (13.5-31.3). The eCVR was high (> 20%) in 738 (53.4%), intermediate in 202 (14.6%) and low in 442 (32%) patients. Men [25.1(15.4-37.3)] showed a higher eCVR than women [18.8 (12.4-27.9) p < 0.001]. The most common risk factor was high LDL-cholesterol (80.8%), most frequently found in women than in men (p = 0.01). The median of risk factors present was three (2-4) without gender differences. Overall we observed that 60 (4.3%) of our patients had none, 154(11.1%) one, 310 (22.4%) two, 385 (27.9%) three, 300 (21.7%) four, 149 (10.5%) five and six, (2%) six risk factors. A higher eCVR was noted in overweight or obese patients (p = 0.01 for both groups). No association was found between eCVR with age or a specific type of diabetes treatment. A correlation was found between eCVR and duration of diabetes (p < 0.001), BMI (p < 0.001), creatinine (p < 0.001) and triglycerides levels (p < 0.001) but it was not found with HbA1c, fasting blood glucose and post-prandial glucose. A higher eCVR was observed in patients with retinopathy (p < 0.001) and a tendency in patients with microalbuminuria (p = 0.06). Conclusion: our study showed that in this group of Brazilian T2DM the eCVR was correlated with the lipid profile and it was higher in patients with microvascular chronic complications. No correlation was found with glycemic control parameters. These data could explain the failure of intensive glycemic control programs aiming to reduce cardiovascular events observed in some studies.

## Introduction

The prevalence of diabetes is increasing quickly in developing countries because of changes in lifestyle that have occurred in the last decades. According to the estimates provided by the World Health Organization (WHO), by the year 2025 more than 75% of people with diabetes will be living in developing countries [1]. The majority of people with diabetes in these countries will be aged between 45-65 years, that corresponds to the most productive period of their lives. The last demographic census in Brazil has estimated a population of approximately 170 million people [2] with many people between 45-65 years. The prevalence of diabetes was estimated to be of 7.6% in a multicenter study conducted in 1988 [3]. Although WHO has estimated a prevalence of diabetes in our country of 7.2% by the year of 2025, more recent data, though scarce and limited to some regions, showed a prevalence of 12.5% in 1999 [4]. According to Brazilian National Data Survey, diabetes is the fifth cause for hospitalization and is one of the ten major causes of mortality in this country [5].

Lately, a better management of the disease has resulted in a longer survival of patients with type 2 diabetes and in a rising prevalence of chronic complications, mainly those cardiovascular [6]. According to different studies the leading cause of death in more than 50% of these patients will be coronary artery disease [7]. Patients with type 2 diabetes have a two to six-fold higher incidence of coronary artery disease and stroke and are considered as having a risk equivalent of a non-diabetic patient with pre-existing heart disease [8]. Moreover, the prognosis of patients with diabetes who have a cardiovascular disease is much worse. The economic impact of the above -mentioned facts will have implications in National Health Care Systems increasing the direct and indirect costs of the disease [9]. It is estimated that 85% of the costs with complications resulting from type 2 diabetes are related to cardiovascular disease [9].

The most well known methods to identify cardiovascular risk are the mathematical equations developed by the Framingham heart study. This study has established a score for prediction of the risk for CVD in 10 years using simple clinical and laboratory variables [10].

The purpose of our study was to stratify the cardiovascular risk in a population of type 2 diabetic patients according to the Framingham prediction equations and to determine if there is a geographical variation in our country. The secondary purpose was to evaluate the association between cardiovascular risk and metabolic and clinical parameters of diabetes control.

## Research Design and Methods

This cross-sectional multicenter study was conducted between May 2000 and May 2001 in thirteen public specialty clinics (diabetes/endocrinology) from eight Brazilian cities. Each clinic has provided data from at least 100 consecutive regularly attending out-patients with type 2 diabetes. We limited our study to patients who had at least one visit yearly, mainly in the last six months and who have been followed at each center for at least one year. For each patient, data for the most recent clinic visit were collected using standardized chart review forms. A clinical visit was defined as a visit with physical examination by a physician. Patients were considered to have type 2 diabetes when it was diagnosed with an age ≥ 30 years, without insulin use in the first year after the diagnosis and without history of ketosis or ketonuria. The following variables were assessed from the most recent clinical visit: age, duration of diabetes, BMI, blood pressure (systolic and diastolic), HBA1c, fasting and post-prandial glucose, total cholesterol, HDL-cholesterol, LDL-cholesterol, triglycerides and therapeutic regimen of diabetes. For each patient we recorded if funduscopy, microalbuminuria and foot examination were done in the prior year or at the most recent clinical visit as recommended by Brazilian Diabetes Society. For this study, five antidiabetic therapeutic regimens were defined: diet, oral antidiabetic drugs, combination of oral antidiabetic drugs, combination of insulin plus oral antidiabetic drugs and insulin as monotherapy.

Sex -specific prediction equations for cardiovascular disease were calculated according to age, diabetes, smoking status, blood pressure (JNC-V categories) and total cholesterol and LDL-cholesterol (NCEP ATP III categories). The 10-year risk of developing coronary heart disease (CHD) was estimated by the prediction equations described by Wilson et al [10] LDL equations were preferably used; when patients missed LDL data, we used total cholesterol equations instead.

When triglycerides were ≤ 400 mg/dL the concentration of LDL cholesterol was estimated indirectly by use of Friedwald formula [11].

We used the recommendations defined by BDS to assess if our patients reached the goals for metabolic and clinical control of diabetes [12].

This study was approved by local ethics committee.

Categorical variables were summarized as count and percentage, and numerical variables as median and inter-quartile range. The Kruskal-Wallis and Mann-Whitney U tests were used for comparisons between groups of variables not normally distributed and the Student t-test and ANOVA were used for the other comparisons. The Chi-square test with Yates correction were used for comparison of categorical variables; the correlation between numerical variables was tested with the Spearman's correlation (rho). Stepwise multivariate regression analysis was done with estimated Framingham risk as dependent variable and variables with p < 0.1 in Spermann's correlation. Statistical analyses were performed with Stata version 10 (StataCorp, 2007).

## Results

Data from 1382 (n = 815; 59.0% female) were analyzed. Median and inter-quartile range of age and duration of diabetes were 57.4 (51-65) and 8.8 (3-13) years, respectively, without differences according to gender The baseline data of the patients are described in table 1. All patients received health care from National Brazilian Health Care System (NBHCS) and most had a low income and education level, similar as in another previous Brazilian survey [12]. Two-thirds of the patients were overweight (42.3%) or obese (35.4%). Women had a significantly higher BMI [28.8 (25.6-32.2 kg/m^2^) than men (27.7 (25-30.1) kg/m^2^) p < 0.001] and a higher prevalence of obesity (41.1.0% vs. 27.2%, p < 0.001).

**Table 1:** Baseline data of the patients

**Variable**	
**N**	1382

**Gender (female) n(%)**	815(59)

**Age (years)**	57.4 ± 9.4

**Diabetes duration (years)**	8.8 ± 7.2

**HbA**_1 _**or HbA**_1_**c (%)**	8.7 ± 2.2

**BMI(kg/m**^2^**)**	28.6 ± 5.1

**FPG (mg/dL)**	168.3 ± 71.2

**PPPG (mg/dL)**	199.8 _+ _88.6

**sBP (mm Hg)**	140.7 ± 22.8

**dBP (mm Hg)**	84.4 ± 11.3

**Total cholesterol (mg/dL)**	211.3 ± 42.8

**Triglycerides (mg/dL)**	177.5 ± 125.2

**HDL(mg/dL)**	44.7 ± 12.6

LDL cholesterol (mg/dL) *	130 ± 38.8

Retinopathy(yes/no) **	551/497

**Albumin Excretion rate (ug/min)**	20 (0.03-4580)

Data are means ± SD, n (%). sBP, systolic blood pressure; dBP, diastolic blood pressure; BMI, body mass index; FPG, fasting blood glucose; PPG, postprandial glucose; total cholesterol;* LDL was not calculated if triglycerides > 4.5 mmol/l(n = 83); **n = 1058

The therapeutic prescription by the time of the last clinic visit was not documented in 208(15%) of the clinical files. Diet alone n = 103(7.5%), one OHA n = 399 (28.9%), OHA combination therapy n = 234 (16.9%), OHA plus insulin = 278 (20.1%) and insulin as monotherapy n = 160 (11.6%) were the used therapeutic regimens.

**Figure 1:**
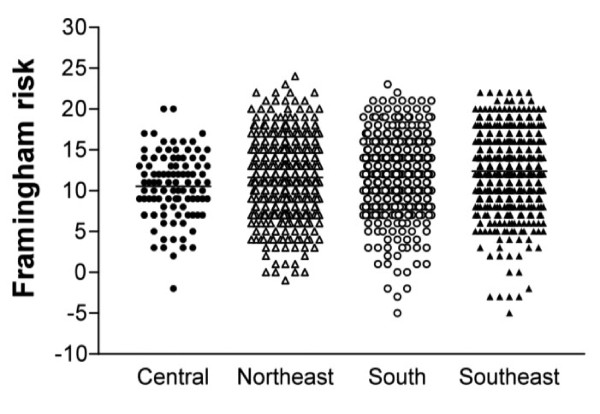
**Estimated Framingham risk according to gender**.

The overall estimated risk was 21.4 (13.5-31.3) 95% CI (20.4-22.2) figure 1. A high risk (> 20%) was found in 738 patients (53.4%), a intermediate risk in 202 (14.6%) and a low risk in 442 (32%). More men than women [360 (63.5%) vs 378 (46.4%) p < 0.001] were classified as being at high risk. Men had a higher estimated risk than women, respectively [25.1(15.4-37.3) vs 18.8 (12.4-27.9) p < 0.001] figure 1. Among patients the most common risk factor was high LDL-cholesterol (80.8%) with a higher frequency in women than men ([687(83.1%) vs 440 (77.6%) p = 0.01]. The median of the number of risk factors present was three (2-4) without difference between men and women. Finally, we observed that 60 (4.3%) of our patients had no risk factor, 154 (11.1%) had one, 310 (22.4%) had two, 385 (27.9%) had three, 300 (21.7%) had four, 149 (10.5%) had five and six (2%) had six risk factors. This distribution was similar in men and women (p > 0.5).

**Figure 2:**
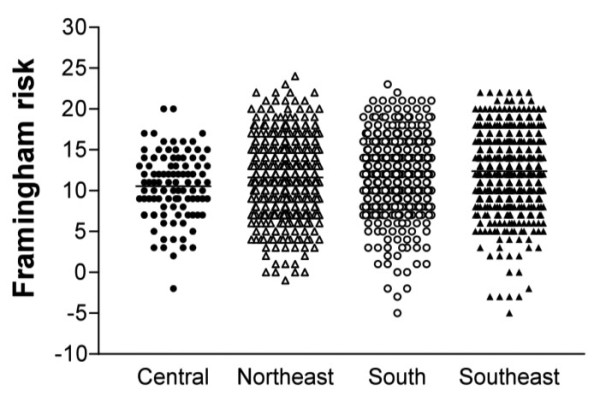
**Estimated Framingham risk according to geographic region**.

A higher estimated risk was noted in overweight or obese patients, respectively [21.4 (14.8-31.3) vs. 20.7 (12.7-30.7) vs kg/m^2 ^p = 0.01). We noted a linear trend between estimated risk and geographic region of the country respectively southwest, northwest, south and central [24.1(15.5-34.0) vs 20.6 (12.3-30.8) vs.19.9 (13.4-30.1) vs. 14.9 (10.9-21.6) p = 0.000] figure 2. No differences were found between estimated risk and age, and type of diabetes treatment. A higher estimated risk was observed in patients with retinopathy, [24.9 (16.3-35.3 vs 18.3(11.4-28.6) % p < 0.001] and a tendency in patients with microalbuminuria, respectively [27.8 (14.6-38.9) vs 22.7(15.7-32.9)% p = 0.06].

A correlation was found between estimated risk and duration of diabetes (rho 0.11 p < 0.001), BMI (rho = 0.11 p < 0.001), creatinine (rho = 0.40 p < 0.001), HDL-cholesterol (rho = - 0.38 p < 0.001) and triglycerides levels (rho = 0.26 p < 0.001). No correlation was found between estimated risk and HBA1c, fasting blood glucose and post-prandial glucose.

In the pooled group a stepwise multiple regression analysis applied to the data with estimated Framingham risk as dependent variable showed HDL-cholesterol, creatinine, gender (male), retinopathy, triglycerides and HbA1c as the significant independent variables. These data are shown in table 2.

**Table 2:** Stepwise multiple regression with estimated Framingham risk as dependent variable in the total population studied

	**Regression analysis**			
	
**Independent variables**	**B**	**Adjusted R**^2^	**95%-CI**	***p *values**
**HDL-cholesterol (mg/dL)**	-0.001	0.14	-0.033 to - 4.17E-04	0.001
**Creatinine (mg/dL)**	0.05	0.21	0.02 to 0.08	0.001
**Gender (male/female)**		0.26	0.03 to 0.09	0.002
**Retinopathy (yes/no)**		0.28	0.006 to 0.04	0.01
**Triglycerides (mg/dL)**	2.15E-04	0.30	6.14 E-05 to 3.7 E-04	0.006
**HbA1c (%)**	-0.006	0.32	-0.01 to -6.3E-04	0.03

As gender and retinopathy were categorical variables the B value can not be calculated

## Discussion

According to our results the majority of our patients with type 2 diabetes are at a high risk of CHD (> 20% in 10 years). Recently a study has compared the performance of Framingham equation and the UKPDS risk engine for the estimated cardiovascular (CV) risk in 10 years [13]. The UKPDS risk engine is a type 2 diabetes specific risk calculator which uses measures of glycemia (HbA1c) and duration of known diabetes as continuous variables in addition to the usual cardiovascular risk factors. Nevertheless, this study has concluded that Framingham equation significantly underestimated the CV risk in 10 years, but only in 2% [13].

We found a high risk and higher means of risk in men in comparison to women. Although some studies have shown a high cardiovascular mortality in women [14,15], a recent meta-analysis with sixteen studies showed that the excess relative risk of cardiovascular (CHD) mortality in women vs men disappeared after adjusting for CHD risk factors, having men still more cardiovascular death attributable to diabetes than women [16]. We observed that women were less likely than men to meet the goals for LDL cholesterol which should focus the attention on gender-specific treatment. Some studies showed similar results and this was mainly related to less aggressive treatment in women [17,18].

We have observed a linear trend between higher estimated risk and geographic regions of our country, however we could not identify which one was responsible for this higher risk (figure 2). Our country has a great racial miscegenation resulting probably in different nutritional habits and different behaviors related to physical activities, smoking and other habits not yet identified. All the above-mentioned could have influenced in the estimated cardiovascular risk.

Similar to the results found in other studies, [19-21] we found higher estimated cardiovascular risk in patients with microvascular complications defined as categorical variables. The positive correlation with plasma creatinine levels should indirectly reflect an association with low glomerular filtration rate and renal function. Many studies have shown that albuminuria is a significant independent predictor of cardiovascular disease, worsening the prognosis of patients with diabetes [19,20]. We also observed a high estimated cardiovascular risk in patients with retinopathy, a marker of microvascular disease. Recent study has demonstrated a link between retinopathy, with the worsening of cardiac structure and function by echocardiography independent of potential confounding variables [21]. In this study it was also noted a trend between the severity of retinopathy and the decrease of left ventricular ejection fraction.

Overall more than 75% of our sample had ≥ 2 risk factors without difference according to gender. However, considering that diabetes *per se *is an important cardiovascular risk factor we can conclude that more than 90% of our sample had ≥ 2 risk factors. Several studies have shown that diabetic patients have more cardiovascular risk factors than non-diabetic individuals [22-25]. In our study, the most common risk factor was high LDL-cholesterol similar to the results of the Strong Heart Study which is an American Indian population based study [26]. This study concluded that for each 10-mg/dL increase in LDL-cholesterol corresponded to a 12% increase in cardiovascular risk, and a 10-mg/dL decrease in HDL-cholesterol was associated with a 22% increase in cardiovascular risk. This latter information was also observed in our study because HDL-cholesterol was the most important independent variable in the model used for multivariate analysis.

In a previous study, we have demonstrated that the majority of our patients with type 2 diabetes attending public specialty clinics did not met the targets for all the cardiovascular risk factors [25]. Our data showed that there is a gap between the reality of routine diabetes care and the guidelines established by the BDS similar to that observed worldwide.

Considering all the different parameters to evaluate glycemic control (HbA1c, fasting and post-breakfast glucose) our study observed only a weak correlation between HbA1c and the estimated cardiovascular risk. Although different, well done clinical trials have demonstrated definitively that good glycemic control can reduce the risk of microvascular complications in patients with type 1 diabetes as well as with type 2 diabetes [27-29] this is uncertain in relation to cardiovascular disease in patients with type 2 diabetes [28]. Several recent large and long-term trials with patients at relatively high risk failed to demonstrate the effects of intensive glycemic control in comparison to standard one on cardiovascular outcomes [30-32]. Recently it was pointed out that for prevention of cardiovascular disease in patients with diabetes type 2 we have to change from glucocentric to a multifactorial approach. In this context the Steno -2 study which was based in a multifactorial aggressive intervention in different cardiovascular risk factors like hyperglycemia, dyslipidemia and high blood pressure, in patients with type 2 diabetes with albuminuria showed a significant reduction in cardiovascular events that persisted 5 to 6 years after the end of the trial [33].

Some limitations of our study must be discussed. The estimated risk derived from the prediction model used is age adjusted and sex-specific and based in an integration between blood pressure and cholesterol information with a weighting approach. Even though our sample comprised only patients with type 2 diabetes, a population with a well known high risk for cardiovascular disease (CVD), other important variables that increase this risk as family history of CVD, presence of chronic complications of diabetes, were not considered in the model. Another point is that the estimated risk derived from this equation is continuous as probably is usual for most of the patients.

In conclusion, the results of our study showed that in this population of patients with type 2 diabetes the estimated cardiovascular risk was correlated with lipid profile but not with glycemic control parameters. Patients with microvascular chronic complications had a higher estimated cardiovascular risk. These data could explain the failure of intensive glycemic control in reducing cardiovascular events observed in some studies.

## Abbreviations

BMI: body mass index; FBG: fasting blood glucose; HbA_1_/or HbA_1_C: glycated hemoglobin; dBP: diastolic blood pressure; sBP: systolic blood pressure; NBHCS: National Brazilian Health Care System; BDS: Brazilian Diabetes Society.

## Competing interests

The authors declare that they have no competing interests.

## Authors' contributions

MBG: has written the manuscript and did the statistical analysis. SAD: has correct the manuscript. DGN, MF, MT, RMF, RRR, GM^7^, JMF, HS, AVB, SC, NR, HP: have collected the data. All authors read and approved the final manuscript.
